# Emerging sports as public health infrastructure: policy transfer lessons from US pickleball to China

**DOI:** 10.3389/fpubh.2026.1753253

**Published:** 2026-03-23

**Authors:** Zhang Weidong, Zheng Wenqiang, Muhammad Arif

**Affiliations:** 1 School of Outdoor Sports, Guilin Tourism University, Guilin, China; 2 School of Tourism Ecology and Environment, Guilin Tourism University, Guilin, China; 3 Guangxi Cultural Tourism and Wellness Integrated Development Center, Guilin Tourism University, Guilin, China

**Keywords:** active aging, adaptive policy and measurement, built environment and health, community-based physical activity, governance and system integration, pickleball, policy transfer and localization, public health policy

## Abstract

Population-level physical inactivity remains a persistent public health challenge, yet many low-threshold activity innovations fail to achieve institutional integration within health and sports governance systems. Emerging sports often diffuse rapidly through grassroots participation without coordinated governance, standardized participation metrics, or aligned facility planning. This institutional gap limits sustained preventative health impact. Using pickleball as a comparative policy case, this study analyzed how governance mechanisms enabled scalable integration in the United States and assessed their transferability within China’s state-led system. Using national participation reports, policy documents, and secondary literature, the analysis identified four defining features of the US model: association-led governance with formal monitoring, a dual-track facility strategy combining retrofitting with selective new construction, community-embedded participation across age cohorts and social settings, and a multi-level competition system linked to the health, tourism, and education sectors. By contrast, diffusion in China is constrained by the absence of unified governance, lack of participation and facility data, insufficient spatial provisions, low public awareness, and weak cross-sector coordination. Based on these contrasts, the study proposes a localization framework that prioritizes phased institutionalization, data-driven planning, low-cost spatial adaptation, educational and community embedding, and incremental integration into public health and tourism programs. The findings suggest that international experience is transferable when functional mechanisms, rather than institutional forms, are adapted to domestic governance conditions. The study provides policy-relevant guidance for incorporating emerging sports into China’s national fitness and population-health strategies, with implications for other emerging sports in state-led governance systems.

## 1 Introduction

Globally, the search for low-cost and socially inclusive forms of physical activity has become central to population-health agendas (1). Countries facing rapid population aging, urban densification, and rising chronic diseases increasingly treat recreational sport as a public-health instrument rather than a leisure commodity (2). Yet the diffusion of novel sports remains highly uneven across governance contexts, even when their physical, financial, and social thresholds for participation are minimal (3). Emerging sports refer to recreational or competitive physical activities that demonstrate rapid participation growth, low entry barriers in terms of equipment and skill acquisition, and incomplete institutionalization within existing governance, facility planning, and public health monitoring systems. Pickleball (a hybrid of tennis, badminton, and table tennis) exemplifies this paradox (4). It requires little space, modest equipment, and minimal skill acquisition and offers moderate-intensity exercise suitable for multiple age groups (5, 6). Despite these enabling attributes, its global spread has diverged sharply (7, 8). In the United States, pickleball has changed from a local hobby to a nationally recognized sport with formal associations, statistical tracking, and community involvement (9). By 2024, it was expected to have nearly 20 million players (10). In China, diffusion has remained slow and fragmented despite dense urban populations, sustained state endorsement of “National Fitness” and “Healthy China” initiatives, and an extensive public sport infrastructure (11). This contrast warrants a comparative inquiry not limited to sport management but extended to how governance architectures enable or constrain health-promoting innovations (12).

Across the globe, emerging and modified sport formats have demonstrated accelerated participation growth, particularly in low-threshold, space-efficient activities. For example, the International Tennis Federation reports sustained expansion of padel participation across Europe and Latin America over the past decade, driven by its accessibility and small-court design (13). Similarly, global recreational running participation has expanded significantly, with large-scale community events and club-based models contributing to increased engagement across North America, Europe, Australia, and parts of Asia (14). In the United States, outdoor participation data indicate substantial increases in alternative and modified sports formats following the COVID-19 period, reflecting demand for socially adaptable, low-entry physical activities (15). These patterns suggest that the diffusion of accessible sports is increasingly common; however, rapid participation growth does not automatically translate into institutional integration within public health and governance systems.

From a public-health standpoint, the “diffusion paradox: the rapid grassroots expansion of a low-threshold physical activity alongside delayed or incomplete institutional integration within formal governance, facility planning, and public health systems” embodied by pickleball demonstrates how institutional design determines whether a physical-activity innovation scales to the population level (16, 17). Policy-transfer theory holds that the successful adoption of foreign models depends less on the intrinsic quality of the innovation than on the fit between the imported mechanism and domestic institutional logic (18). Diffusion-of-innovation theory posits that adoption is contingent upon perceived advantages, compatibility, and observability within existing social systems (19). In contrast, institutional theory asserts that adoption necessitates normative and regulatory legitimacy, rather than mere awareness (20). When governance frameworks, incentive structures, and social norms diverge, for instance between the decentralized US model and China’s state-directed policy system, the same innovation may follow contrasting trajectories (21). Consequently, pickleball’s limited diffusion in China reflects not a lack of resources or cultural interest but a misalignment of institutional conditions that structure incentives, authority, and cross-sector coordination. Existing research on pickleball focuses primarily on micro-level determinants such as motivation, enjoyment, or skill acquisition (22, 23), while macro-level governance and policy dynamics remain underexplored. Chinese scholarship emphasizes promotional narratives and instructional practices rather than structural diagnostics. Few studies treat pickleball as a policy-transfer case relevant to comparative governance or public health systems (24, 25). Likewise, research on sport globalization continues to privilege elite or professional domains, overlooking how low-threshold, health-oriented sports become embedded in national fitness architectures. This omission is non-trivial because such sports yield the highest marginal public-health returns (26, 27): they mobilize previously inactive adults, facilitate intergenerational activity, and demand minimal infrastructure investment.

Bringing sport governance into dialog with population-health research offers dual analytical value (28, 29). First, it connects health equity to sports participation by showing how accessibility and cost affect who benefits on a large scale (30). Second, it reframes sport policy as a lever within preventive and community-based health systems, rather than as an extension of competitive or clinical domains (31). This study adopts this lens, using pickleball differential diffusion as a case to show how institutional configurations mediate the health-promotion potential of recreational innovations. Understanding these mechanisms is policy-relevant for governments seeking to expand participation without imposing heavy fiscal burdens. Pickleball is selected as an analytically appropriate case for policy transfer because it combines low equipment costs, moderate spatial requirements, simplified skill acquisition, and strong intergenerational appeal. These characteristics reduce cultural and economic barriers to adoption, allowing governance and institutional design factors to emerge as primary determinants of scaling success. Policy transfer and diffusion theories clarify how institutional mechanisms shape adoption trajectories (32, 33). Imported models seldom transfer intact; they necessitate reinterpretation and restructuring within domestic administrative and cultural limitations. The US model, which is mostly self-organized through associations, depends on decentralized decision-making, clear monitoring, and the ability to change facilities as needed (34). China’s sports system is state-centric and plan-driven, emphasizing administrative authorization and coordinated implementation. Examining how a loosely institutionalized model interacts with a bureaucratic governance environment reveals the conditions under which foreign policy ideas are translatable rather than transferable. Taken together, these institutional conditions help explain why favorable demographic and spatial factors alone have not translated into rapid diffusion within China’s governance context.

Experiences in other Asian contexts further suggest that institutional configurations significantly influence sport innovation adoption. In Japan, national sport policy reforms and municipal-level programming under the Sport Basic Plan have emphasized lifelong participation and school–community integration as central mechanisms for activity diffusion (35). Similarly, South Korea’s community-based sport club expansion under the Ministry of Culture, Sports and Tourism has strengthened local participation infrastructures within dense urban environments (36). Singapore’s centralized sport governance model, led by Sport Singapore, has supported the structured diffusion of emerging recreational activities through coordinated facility development and nationwide participation campaigns (37). These cases suggest that diffusion trajectories across Asian contexts are strongly mediated by governance coordination, spatial planning capacity, and policy alignment rather than cultural receptivity alone. This reinforces the institutional emphasis of the present analysis.

Diffusion-of-innovation theory complements this by illuminating the social mechanisms of uptake (19). In the US, pickleball spread through visible community practice in parks and schools, enhancing observability and trialability (38, 39). In China, diffusion depends more heavily on formal recognition; activities not legitimized by state endorsement typically remain peripheral. This asymmetry aligns with the institutional-isomorphism logic, wherein organizations emulate practices that confer legitimacy rather than efficiency. Until pickleball attains normative validation within China’s sport-administration framework, diffusion may likely remain constrained irrespective of the latent social demand. These theoretical insights converge on a central proposition: the health impact of emerging physical activities depends as much on institutional legitimacy as on individual motivation (7, 8). From a public-health standpoint, the implications are becoming increasingly significant (40). Sedentary behavior is now one of the primary risk factors for non-communicable diseases (41). Older people need low-impact, socially engaged activities that keep both their bodies and minds active (42, 43). Pickleball meets these needs because it encourages active aging and social interaction and can be played in small urban and institutional spaces (44). Realizing this potential, however, requires coordinated policy design, including participation monitoring, facility standards, and cross-sector partnerships linking sport, health, and planning agencies (45). The gap between pickleball’s recognized health suitability and its limited institutionalization in China exemplifies the broader difficulty of translating individual-level activity innovations into structural public-health assets.

This study addresses three interrelated knowledge gaps. First, comparative sport-policy research rarely examines the transfer of recreational sports despite their disproportionate public-health benefits. By analyzing pickleball, the study extends policy-transfer theory into the domain of preventative-health governance. Second, China provides a counterfactual setting for theory evaluation, enabling analysis of policy mobility within a centralized, state-led governance system. Third, the paper integrates sport sociology with public-health governance by framing pickleball not merely as a leisure activity but as a social technology that fosters intergenerational exchange, social capital accumulation, and psychological resilience—well-established determinants of population health. This study employs a structured comparative policy analysis to examine how emerging sports become institutionally embedded within public health systems. The analysis draws on publicly available national participation reports, sport governance documents, policy frameworks, municipal planning records, and peer-reviewed secondary literature published between 2018 and 2025. Documents were selected based on their relevance to participation measurement, governance coordination, facility adaptation strategies, and cross-sector policy integration. Materials were included only if they had policy relevance and did not focus exclusively on technical coaching or elite performance. The comparative framework assesses how institutional mechanisms that have facilitated scalable integration in the United States operate within, and require adaptation to, China’s state-led sport and public health governance architecture. The objective is functional transfer evaluation rather than institutional replication. Section 2 reconstructs the US governance architecture, emphasizing association leadership, data-driven planning, facility strategies, and cross-sector embedding. Section 3 diagnoses China’s structural bottlenecks, including fragmented authorities, absent data infrastructure, venue scarcity, and low youth penetration. Section 4 proposes a localization framework structured around five functional pathways: unified governance, spatial optimization, multi-level competition and talent systems, cross-sector integration (“pickleball + health/education/tourism”), and data-driven collaborative governance. Section 5 discusses the implications for policy transfer and health-system design, and Section 6 concludes with strategic recommendations for sustainable institutionalization.

## 2 Development logic and governance architecture of US pickleball

Understanding the institutional foundations that enabled pickleball to scale in the US is essential for public-health analysis, because it reveals how a low-threshold activity can be structurally translated into widespread physical-activity practice at the population level.

### 2.1 Policy support and association governance

The contemporary expansion of pickleball in the US emerged from a governance arrangement in which public authorities, civil associations, and community institutions interacted without centralized command. USA Pickleball (USAPA), initially a volunteer network, evolved into a nationally recognized standard-setting body responsible for rule codification, coach accreditation, facility certification, and competition oversight (12, 46). Its collaboration with the Sports & Fitness Industry Association (SFIA) established regular data reporting on participation and demographics, converting a recreational fad into a statistically observable phenomenon. Municipal governments used this evidence to classify pickleball as a legitimate component of community fitness programming rather than an informal pastime (5). The federal government did not directly manage pickleball but created an enabling environment through physical-activity promotion agendas led by agencies such as the President’s Council on Sports, Fitness & Nutrition. States and municipalities integrated pickleball into park and recreation policies, school curricula, and senior-fitness initiatives (7, 8, 27). This alignment between civil governance and the public mandate generated legitimacy without bureaucratic gatekeepers. In population-health terms, association-led governance lowered procedural inertia and expedited the institutionalization of an activity capable of mobilizing inactive adults into regular movement (3, 29).

### 2.2 Facility supplies and spatial development strategies

Pickleball’s rapid expansion was also driven by a facility strategy that privileged low-cost adaptation over capital-intensive construction (11, 16). Local governments implemented extensive retrofitting policies, enabling the conversion of existing tennis and basketball courts with additional line markings and modular nets. This strategy permitted rapid spatial diffusion into neighborhoods without requiring new land acquisition or construction budgets (25, 47). By embedding courts in schools, parks, and community centers, municipalities integrated physical-activity infrastructure into daily living environments rather than confining it to specialized venues (9). Purpose-built facilities emerged later, primarily when participation reached a threshold justifying tournament hosting or structured instruction. These venues supported competitive and instructional functions, but diffusion momentum had already been established through low-cost spatial penetration. This sequence shows how public health works: infrastructure is built first for everyone to use and then for specific groups (4).

Municipal practice further illustrates this logic. For example, the Seattle Parks and Recreation Department implemented a phased conversion of underutilized tennis courts into shared pickleball facilities within neighborhood parks, responding to participation growth while minimizing capital expenditures. Rather than constructing specialized venues at the outset, the city prioritized low-cost striping and modular net systems embedded in community settings, thereby expanding immediate access while collecting usage data to inform long-term planning decisions. Fast-growing metropolitan areas like Austin have also implemented similar adaptive strategies, where incremental retrofitting preceded selective development of purpose-built tournament facilities. These cases demonstrate how local governments operationalize adaptive reuse as a pragmatic public-health infrastructure strategy rather than a speculative investment model. Local governments have lowered geographical and financial barriers to entry by putting adaptive reuse and spatial embedding at the top of their priorities list in residential catchments. This approach enabled sustained participation among older adults, cost-constrained households, and time-limited workers, three key populations central to chronic-disease prevention agendas (2, 48).

**Figure 1:**
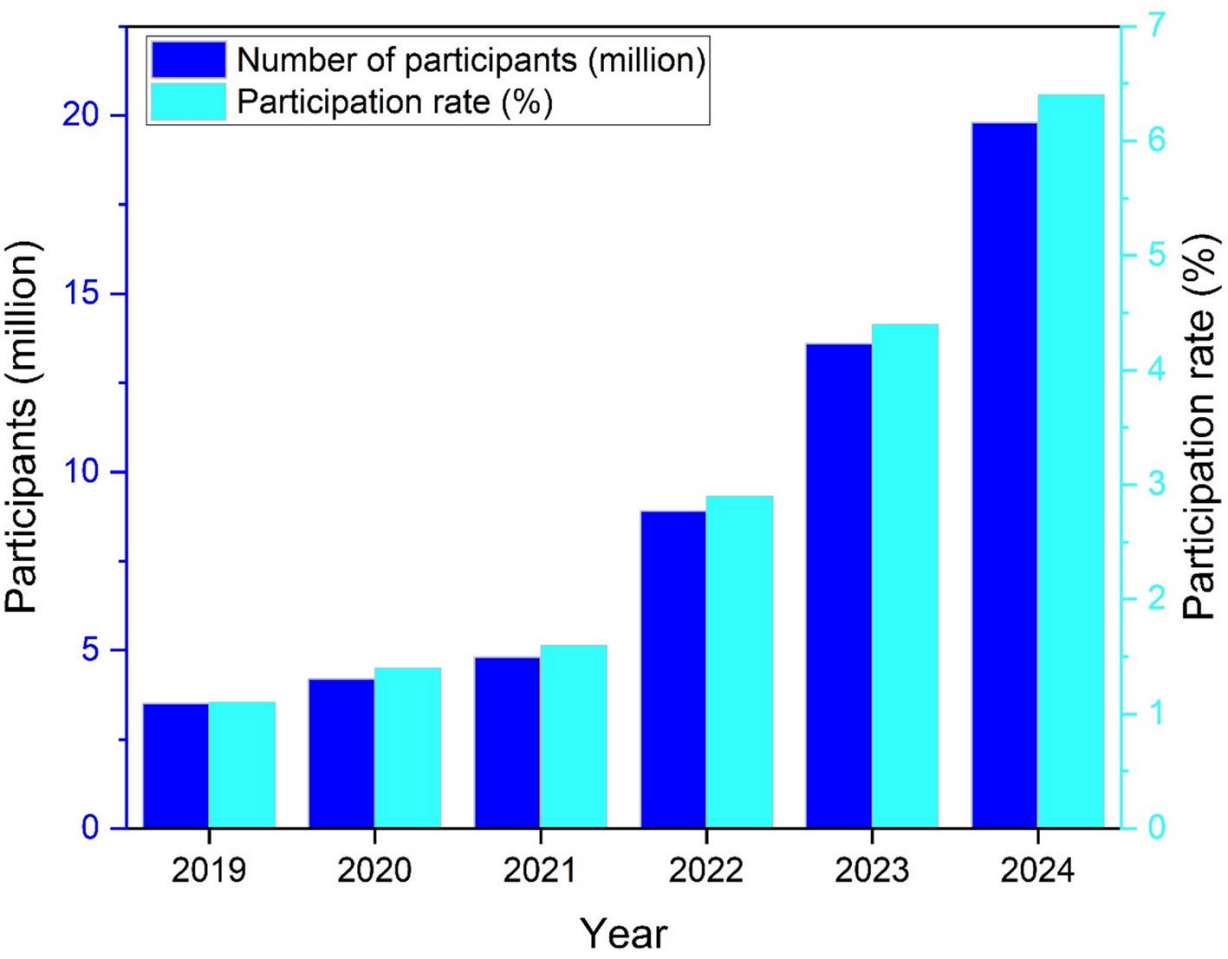
Growth in total pickleball participation and participation rate in the United States, 2019–2024.

Beyond fiscal efficiency, adaptive retrofitting carries significant equity implications. By converting underutilized courts within existing neighborhood park systems and school grounds, municipalities expand access without concentrating investment in high-income districts or newly developed zones. Because retrofitted courts are embedded within residential catchments, they reduce travel distance, lower entry costs, and integrate participation opportunities into daily routines. From a public health perspective, such spatial proximity disproportionately benefits older adults, time-constrained workers, and households with limited disposable income, which are often underrepresented in structured sport participation. However, infrastructure provision alone does not guarantee equitable outcomes; inclusive scheduling policies, open-access regulations, and community programming are required to translate spatial accessibility into sustained and demographically balanced participation. Retrofitting, therefore, functions as an enabling condition for health equity rather than as a direct determinant of population health improvement. US participation has grown exponentially in the past 5 years, reflecting structural diffusion rather than episodic popularity. Figure 1 illustrates the sustained upward trajectory of pickleball participation in the US. The consistent annual increase suggests that adoption has moved beyond an early-adopter phase and entered a rapid diffusion stage. Such momentum typically occurs only when governance, facilities, and social acceptability align. This trend provides the empirical justification for treating pickleball as a mature and transferable policy case rather than an emerging or unstable phenomenon.

### 2.3 Participation demographics and community embedding

Participation data for 2024 indicate that pickleball adoption in the US spans age, income, and ethnic groups in patterns consistent with broad-based diffusion (Table 1). Age-stratified participation rates offer an indirect insight into institutional embedding. Youth participation reached 7.8% among children aged 6–12 and 8.4% among adolescents aged 13–17, both exceeding proportional representation benchmarks (Index > 120). These figures are consistent with exposure to school-based physical education and youth recreation programming. Participation among adults aged 55–64 and 65 + exceeded 5 % of subgroup populations, involving nearly 3 million older participants nationally. While consolidated administrative statistics on senior-centered adoption are not centrally reported, this demographic concentration aligns with documented integration into active-aging and community recreation initiatives (42, 44). These age-specific rates do not directly measure institutional adoption but serve as structural indicators of embedding within educational and senior-service contexts.

**Table 1:** Demographic distribution of pickleball participants in the United States, 2024.

Category	Subgroup	Participants (000 s)	Participation rate (%)	Index (vs. total population)
Sex	Male	11,604	7.6	119
	Female	8,203	5.2	81
Age	6–12	2,220	7.8	121
	13–17	1,810	8.4	132
	18–24	2,808	9.5	149
	55–64	2,316	5.3	83
	65+	2,993	5.3	83
Income	≥ USD 100,000	8,756	8.5	133
Education	Postgraduate degree	3,844	9.1	142
Race/Ethnicity	Hispanic	3,039	7.4	115
	Asian/Pacific Islander	1,237	7.6	119

Participation rate refers to the percentage of individuals within each subgroup who reported participating in pickleball during the reporting year. The index is calculated as: Index = (Subgroup participation rate ÷ Total US population participation rate) × 100. An index value of 100 indicates proportional representation relative to the overall population; values above 100 indicate above-average representation, and values below 100 indicate below-average representation. Data derived from the (51) Participation Report.

**Figure 2:**
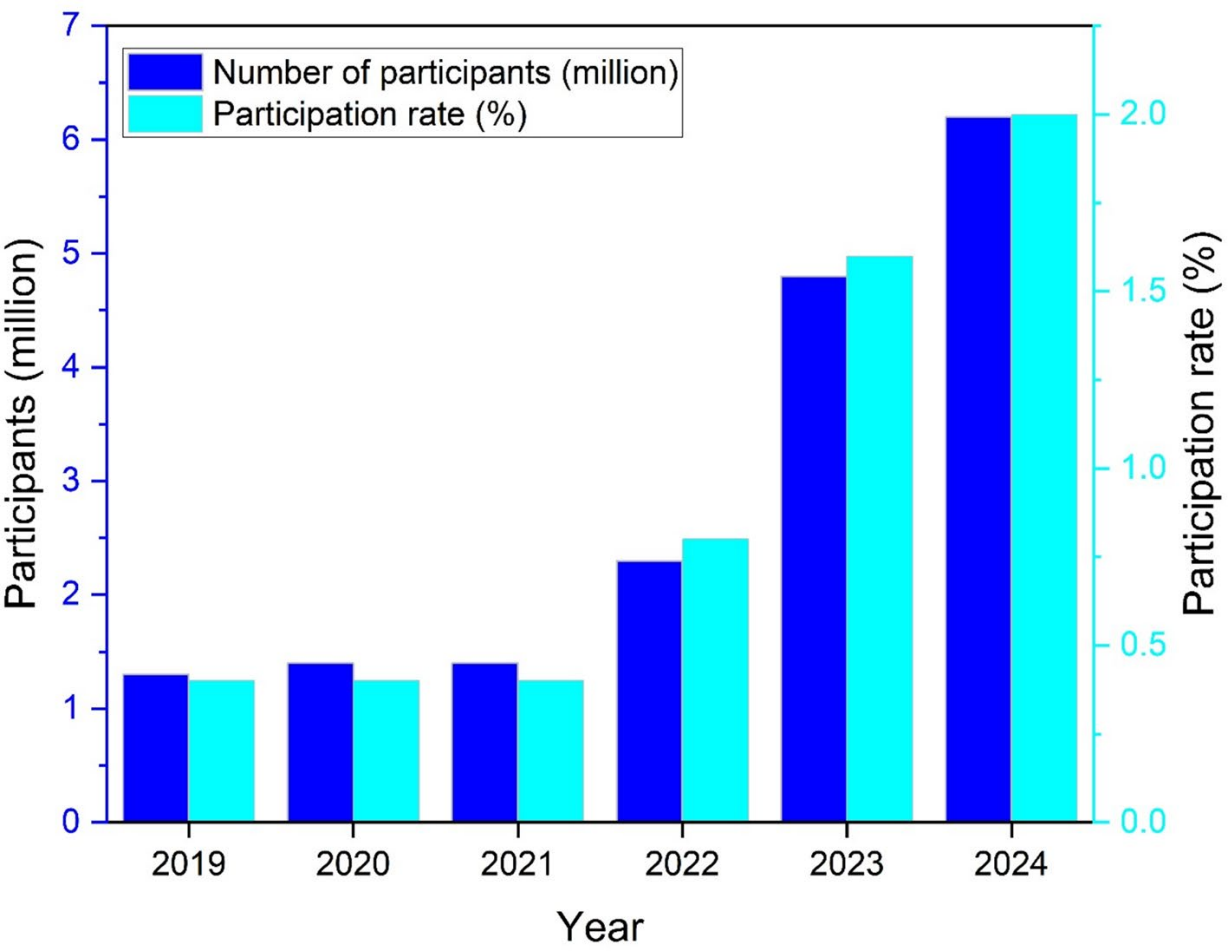
Growth in core pickleball participation and participation rate in the United States, 2019–2024.

Figure 2 further demonstrates that core participation is distributed across all age groups rather than confined to a single demographic segment. The presence of stable core users is widely interpreted in diffusion research as a signal of institutional consolidation rather than episodic popularity (49). These patterns reinforce the interpretation that pickleball has transitioned into a durable component of local recreation systems. Institutional embedding is observable at the municipal and school levels. In cities such as Seattle and Austin, public school districts and parks departments have coordinated introductory programming within physical education curricula and community recreation initiatives. In Washington State, selected districts have incorporated pickleball modules into middle-school physical education, supported by teacher-training workshops and starter equipment grants. In Austin, community centers have partnered with local clubs to host youth clinics and intergenerational open-play sessions in neighborhood parks. These arrangements illustrate how municipal governance and educational institutions jointly normalize participation and lower entry barriers during formative years.

Middle-aged and older adults engaged due to the sport’s moderate intensity, low joint impact, and social format, features associated with sustained physical activity adherence (1, 42). Early diffusion was more pronounced among resource-advantaged groups, particularly high-income and college-educated participants, but the sport’s low equipment cost and neighborhood-level facility access facilitated downstream adoption across diverse communities. Embedding in institutional settings enhances behavioral stability. Schools, senior centers, clubs, and community organizations provide recurring participation opportunities rather than one-time exposure. Local clubs coordinate instruction and informal competition, supplementing municipal capacity and reducing fiscal dependence. In public-health terms, sustained participation depends less on awareness than on routine access supported by institutional anchors (28, 50).

The participation data are drawn from nationally administered survey instruments using large-scale household sampling designed to approximate demographic representativeness across the US (51). As with most participation surveys, the dataset relies on self-reported activity and may be subject to recall bias or reporting inflation. The data capture participation prevalence rather than frequency, duration, or clinical health outcomes. Accordingly, they are used descriptively to illustrate adoption patterns and demographic reach rather than to estimate causal health effects.

### 2.4 Competition system and cultural integration

The US developed a structured competition ladder that linked recreational participation with organized events without erecting exclusionary barriers. Local leagues, school tournaments, and community events created progression pathways that retained participants and diversified motivations for continued play. Standardized rules, officiating, and accreditation supported interoperability between the local and national systems (17, 52). Exposure to collegiate and senior-division events normalized pickleball within a formal sport culture, strengthening its legitimacy among institutions responsible for health-promotion programming (31, 44). Cultural integration further reinforced the uptake (33). Workplaces, faith communities, veterans’ organizations, and retirement communities institutionalized pickleball as a medium of social interaction and psychosocial support. Integration into lifestyle and wellness events increased visibility while reinforcing normative approval. These processes expanded the social rewards attached to participation, a known determinant of long-term physical activity adherence (45). For population-health governance, the US case shows that competitive structures and cultural embedding serve not to professionalize the sport but to stabilize participation as a sustained health behavior.

Rapid expansion has generated governance challenges. In several US cities, retrofitting tennis courts triggered user conflicts and scheduling disputes, requiring revised allocation policies. The sport’s acoustic profile also led to neighborhood noise complaints in dense areas, prompting time restrictions and mitigation measures (53). Injury surveillance reports increased emergency-department visits, particularly among older adults, including fractures and sprains (40, 54). These dynamics underscore the need for regulatory adaptation alongside expansion. As indicated in Table 2, pickleball functions predominantly as a complementary activity within the US physical-activity portfolio, with high co-participation in treadmill exercise, walking, bowling, and free-weight training. The exceptionally strong overlap with tennis participation implies that racquet-sport literacy significantly reduces adoption barriers. In China, where badminton and table tennis are very popular, these results suggest that existing racquet-sport communities could be good places to start targeting. The additive nature of participation further reinforces the feasibility of embedding pickleball within existing community fitness ecosystems.

**Table 2:** Participation in other physical activities among US pickleball players, 2024.

Activity	Participants (000 s)	Participation rate (%)	Index
Treadmill exercise	8,209	41.4	225
Walking for fitness	7,853	39.6	106
Bowling	7,330	37.0	227
Free weights (dumbbells)	6,351	32.1	176
Tennis	4,953	25.0	300

The index compares activity participation among pickleball players to the total US population. Values >100 indicate that pickleball participants are more likely to engage in that activity.

### 2.5 Synthesis: characteristics of the US model

The US model is characterized by four interdependent features with direct relevance to public health systems (10). First, governance is civil-led but publicly enabled: associations generate rules, data, and coordination, while governments supply legitimacy and integration without central control. This reduces institutional friction and accelerates the translation of recreational innovation into community-level health practices (19). Second, institutionalizing data through annual reporting and longitudinal tracking turns sports participation into something that can be measured. The results let policymakers use evidence instead of advocacy to justify spending money on preventative health (18). Third, infrastructure pragmatism that prioritizes retrofitting and fine-grain spatial embedding lowers costs and expands physical-activity access at the neighborhood scale, aligned with equity and aging-in-place strategies (38). Fourth, multi-scenario social embedding transforms pickleball from optional recreation into a recurring behavioral setting present in schools, workplaces, senior services, and rehabilitation contexts. This process ensures that participation is supported not only by individual motivation but also by institutional scaffolding (20). Together, these elements illustrate how a non-elite, low-threshold sport has become a structural vector for mass participation and community-level health promotion. The US experience demonstrates that the population-health potential of emerging sports depends less on athletic merit than on governance configurations that convert accessibility into institutionalized practice. Importantly, these pillars are transferable because they represent core governance functions rather than context-specific institutional arrangements, meaning that their operational logic can be embedded within different administrative systems without structural imitation.

## 3 Diagnostic analysis of China’s current stage and structural bottlenecks

Diagnosing China’s current stage of pickleball development is essential for understanding how institutional gaps and administrative fragmentation constrain equitable access to emerging health-promoting physical activities.

### 3.1 Absence of unified governance and organizational authority

China’s pickleball development remains formative and fragmented. No single national governing body or administrative division under the General Administration of Sport (GAS) assumes full responsibility for promotion, rule standardization, or event oversight. Community clubs, school sports departments, and retired athletes’ associations spread the word. These entities sustain local enthusiasm but operate in isolation, producing uneven technical standards, certification procedures, and participation monitoring. In China’s highly centralized sports system, the absence of a nationally recognized authority limits access to state subsidies, facility scheduling, and participation in official policy frameworks like the National Fitness Plan. Without institutional endorsement (55), pickleball lacks bureaucratic legitimacy and fiscal continuity. Local promoters depend on ad-hoc sponsorship and volunteer support, making expansion unstable (11). In Zhejiang and Guangdong, provincial efforts have been made to set up pickleball committees within existing racquet-sport federations. However, the lack of vertical coordination makes it impossible to plan together. This governance vacuum limits sport’s capacity to contribute to public-health objectives. In systems where administrative approval decides who can get funding and use public facilities, unrecognized sports stay on the outside, no matter how much people want them. The absence of a coherent national association therefore weakens institutional trust (56), constrains data integration, and obstructs strategic alignment required to deliver low-cost physical-activity options for aging and urban populations. In practice, emerging sport promotion in China often relies on localized pilot activities initiated by municipal or provincial sport bureaus under broad national fitness directives. However, without a formally designated national federation, such initiatives tend to remain fragmented and lack sustained vertical coordination.

### 3.2 Lack of data systems and evidence-based policy foundation

A second structural bottleneck is the absence of standardized data systems. While the US has longitudinal participation databases maintained by national associations, China lacks an official platform for documenting player numbers, venue distribution, or demographic composition. Available figures come from fragmented sources like social media records, club newsletters, or small regional surveys (25). For example, participation information is often dispersed across commercial court booking platforms, privately organized club registries, and locally reported tournament announcements, none of which are integrated into a centralized national sport governance database. These sources offer no reliable baseline for policy design or resource allocation (49). Data scarcity prevents the sport authorities from recognizing pickleball as a measurable component of the national fitness landscape (2). Municipal sport bureaus allocate budgets using quantified indicators such as registered participation rates and facility coverage. When an activity lacks officially verified participation and venue statistics, it cannot enter these allocation formulas and therefore receives no dedicated fiscal recognition. Consequently, resource distribution defaults to established sports with stable statistical visibility, including basketball, badminton, and table tennis. When no statistics exist, emerging sports are invisible within their planning algorithms (27, 31). As a result, legacy sports such as basketball, badminton, and table tennis continue to receive fiscal support. The absence of credible data also impedes public health evaluations (22). Without participation monitoring, it is impossible to assess how pickleball contributes to physical activity targets, gender equity, or the inclusion of older adults. Policymakers cannot model cost–benefit ratios or demonstrate preventative-health savings, leaving pickleball outside the logic of evidence-based governance (3). Establishing a unified data infrastructure would enable the sport to move from anecdotal enthusiasm to policy-relevant evidence, linking participation trends with measurable health indicators.

### 3.3 Facility scarcity and inefficient resource utilization

Spatial and infrastructure constraints remain major barriers to scaling up participation (30). Although the national guidelines encourage schools and public venues to open their facilities to communities, their implementation is uneven and lacks explicit references to pickleball. Most cities have not set technical standards for designing courts, keeping surfaces safe, or managing shared use (57). As a result, local authorities often categorize pickleball as a marginal activity unsuited to large-scale investment. Where facilities do exist, they are frequently improvised conversions to badminton or tennis courts with inconsistent dimensions and inadequate buffer zones. This affects play quality and safety, discouraging sustained participation for beginners (4). Preliminary observations from major metropolitan areas such as Beijing, Shanghai, and Shenzhen indicate uneven levels of institutional visibility and facility integration, with most courts located within multipurpose venues and subject to scheduling competition rather than formally incorporated into municipal sport infrastructure planning. In addition, fragmented property rights across schools, residential committees, and neighborhood centers cause scheduling conflicts and uneven user fees. In several cities, informal reports from community sport centers indicate that proposed pickleball sessions have been reassigned or restricted during peak hours because existing badminton or table tennis programs retain priority designation within shared indoor facilities, illustrating how institutional hierarchy shapes access. These frictions reduce utilization efficiency (33, 39): many potential courts remain idle during off-peak hours. Financial mechanisms exacerbate inequality. Grassroots facility renovation funds are awarded competitively to projects with strong administrative endorsements, disadvantaging independent clubs. Private developers have built pickleball courts in resorts and commercial buildings, but these places charge high prices and cater to wealthy customers. Hourly rental fees in privately operated venues often exceed those of public community facilities by several multiples, particularly during evening and weekend peak periods, creating financial barriers that discourage middle-income and older participants from sustained engagement. The result is a dual system, like undersupplied public courts and exclusive private spaces, that contradicts the egalitarian objectives of the National Fitness Program. From a public-health perspective, spatial scarcity and cost barriers restrict equitable access to a form of exercise particularly suitable for middle-aged and older adults, the very populations most in need of moderate social physical activity (1, 6).

### 3.4 Weak social awareness and youth penetration

Limited public awareness and weak youth participation further constrain diffusion (28). While curiosity about new sports is growing (34, 53), pickleball lacks sustained media exposure or consistent representation in national health campaigns. Most citizens have heard of the sport but cannot identify its rules, benefits, or health value. Television coverage is minimal, and online content remains sporadic. For instance, compared with established racquet sports such as badminton and table tennis, pickleball generates substantially lower levels of mainstream sports media reporting and digital search visibility on national platforms, reflecting its marginal presence in public discourse. Without cultural visibility, pickleball has yet to enter the symbolic repertoire of modern, health-conscious lifestyles. Education, a key channel for long-term behavior change, has not institutionalized the sport. Schools rarely include pickleball in their physical education curricula, and teacher-training programs rarely prepare instructors (12, 58). In contrast, badminton and table tennis achieved rapid youth uptake following early incorporation into school curricula, standardized coaching certification pathways, and sustained representation in national competitions, creating generational continuity and entrenched cultural legitimacy. Pilot experiments in Shanghai and Hangzhou demonstrate strong engagement when the sport is introduced, but these remain isolated efforts reliant on motivated individuals. Universities continue to prioritize traditional racquet sports for curricular familiarity and examination convenience, leaving pickleball on the margins of formal education. This pattern reflects broader cultural and institutional dynamics, including examination-driven curriculum stability, teacher certification pathways oriented toward established sports, and a symbolic hierarchy in which long-recognized disciplines are perceived as more legitimate within formal education systems. The age profile of participants thus skews toward middle-aged and retired citizens, who value moderate intensity and sociability but are less likely to sustain competitive or digitally networked engagement. The sport lacks generational continuity and the ability to innovate without young people getting involved (5). For public-health governance, this imbalance weakens long-term population reach: interventions confined to older cohorts cannot produce cumulative gains in active-lifestyle norms or reduce sedentary risk among younger demographics (17).

### 3.5 Low capacity for cross-sector integration

A fifth structural issue lies in the weak linkages between pickleball and adjacent policy sectors such as health, education, tourism, and technology. Unlike the US, where cross-sector collaboration expanded participation through wellness programs and event tourism (38, 59), China’s policy system remains compartmentalized. Health authorities seldom integrate pickleball into community-exercise prescriptions or rehabilitation services, even though its low-impact characteristics suit cardiac and musculoskeletal patients. Rehabilitation centers rely largely on traditional Tai Chi or stretching exercises, missing the opportunity to diversify offerings for older population. Tourism and commercial agencies have begun exploring pickleball events in resort areas, especially coastal regions, yet these efforts emphasize economic promotion rather than public access. While such initiatives contribute to local employment and visibility, they do not necessarily enhance participation in equity or health outcomes (10, 44). To meet population health goals, tourism-based programs would need community versions that are affordable and open to everyone (40). For example, municipal pilot initiatives in cities such as Shanghai and Shenzhen have experimented with short-term collaborations between community sport centers, local health promotion offices, and schools to introduce introductory pickleball sessions linked to active aging campaigns, although these efforts remain project-based rather than institutionally embedded. Institutionally, the lack of coordination among the General Administration of Sport, the Ministry of Education, and the National Health Commission prevents sharing objectives. This coordination gap is particularly significant given that frameworks such as the Healthy China 2030 Plan, the National Fitness Program, and recent physical education reform directives all emphasize expanding population physical activity and school sport engagement, yet do not explicitly incorporate emerging activities such as pickleball into their implementation guidelines. Each agency operates under separate mandates and budget lines, resulting in siloed initiatives. Because ministerial performance assessments and funding allocations are evaluated on sector-specific indicators rather than shared outcome metrics, agencies face limited administrative incentives to invest resources in jointly governed programs. Without inter-ministerial task forces or pooled funding mechanisms, cross-sector projects would struggle to achieve continuity (7, 8). Digital infrastructure could help close some of these gaps by linking clubs, facilities, and participants through integrated platforms. However, a national system has not yet been implemented. Strengthening cross-sector integration is crucial because it transforms sport from a recreational niche into a structural instrument for social well-being, enabling joint outcomes in health, education, and urban vitality.

### 3.6 Summary of diagnostic findings

Overall, China’s pickleball landscape exhibits grassroots dynamism but systemic fragility. Governance fragmentation, data deficits, and facility scarcity constrain institutionalization; weak social visibility and limited youth penetration undermine sustainability; and cross-sector compartmentalization restricts sport contribution to broader policy goals. These interlinked bottlenecks reflect a developmental stage where local enthusiasm substitutes for national coordination (60). From a governance standpoint, the pattern remains bottom-up, unstandardized, and dependent on short-term initiatives rather than structural design. From a public-health perspective, the implications are twofold (45). First, the absence of cohesive governance prevents community participation, translating into measurable health impacts (9). Second, uneven facility access and demographic concentration produce inequitable benefits, limiting pickleball’s potential to serve as an inclusive, preventative-health platform (7, 8). Nevertheless, the underlying conditions remain favorable. Urban residents increasingly demand affordable, social, and moderate-intensity activities; the aging population requires accessible recreation; and the policy rhetoric emphasizes “sports–health integration.” These dynamics provide latent momentum that, if institutionalized, could transform pickleball into a scalable vehicle for active aging, social cohesion, and preventative-health innovation (19). The following section therefore outlines a localization framework for translating governance experience into China’s policy environment, emphasizing phased institutionalization, data system construction, spatial optimization, and integration with the health, education, and tourism sectors to advance equitable participation and long-term public-health outcomes.

## 4 Experience translation framework: US → China localization pathways

Translating the functional logic behind US pickleball development into China’s governance environment requires identifying mechanisms that are compatible with a state-led, policy-driven fitness system while still enabling bottom-up participation. The goal is not replication but conditional alignment: determining which governance, spatial, and social mechanisms could operate under Chinese institutional constraints to support population-level health promotion.

### 4.1 Institutional and policy governance localization

The US demonstrates that association-led governance, recognized but not controlled by the state, can provide continuity, legitimacy, and operational efficiency. In China’s context, localization would likely require a nationally recognized governing entity housed under or affiliated with the General Administration of Sport (GAS) to consolidate standards, data, event management, and sector coordination (11). Such an entity would not replicate the US civil model but could borrow its functional roles. These could include rule standardization, certification, and monitoring while operating within Chinese administrative hierarchies. Provincial and municipal sub-branches could mirror the US horizontal coordination function, not as autonomous actors but as delegated arms of a unified national authority. Regular publication of a Pickleball Development Report, modeled on US practice, would create an evidentiary foundation for decision-making and enable the sport to enter planning and budgeting processes that depend on measurable outcomes. A public-service outsourcing model, where social organizations run activities under state supervision, could balance state oversight with non-state dynamism (22). In terms of public-health relevance, formal institutional anchoring would unlock structural support for using pickleball as a platform for active aging, chronic-disease prevention, and community fitness rather than isolating it as a volunteer initiative (4).

### 4.2 Facility planning and spatial development localization

The US facility model demonstrates that widespread access was achieved not by building specialized venues first but by low-cost retrofitting of existing courts embedded within daily living spaces (49). For China, localization could follow a similar logic by integrating retrofit projects into ongoing urban renewal and community-fitness schemes. Issuing technical guidelines for shared-use striping, safety buffers, and illumination would reduce administrative uncertainty and encourage local governments to classify pickleball retrofits as compliant public infrastructure rather than unregulated experiments (10). Selective construction of comprehensive venues in demonstration cities would serve dual roles: training and competition provision as well as symbolic anchoring for media communication and institutional recognition (12). Co-ownership models, which include public land with private operating capital, would align with China’s “government guidance + social participation + market assistance” policy on grassroots sports. Embedding pickleball into the “15-minute fitness circle” standard would institutionalize its presence at a neighborhood scale, advancing health equity by reducing travel and cost barriers. In rural areas, modular prefabricated courts located next to older population-activity plazas or village cultural centers could make it easier for people with higher health risks to acquire the services they need (17). Thus, spatial localization would not merely supply facilities but embed a health-enabling environment into the everyday urban and rural fabric.

### 4.3 Community cultivation and participation expansion

In the US, pickleball became durable because participation was socially scaffolded rather than individually motivated. For China, localization would depend on converting scattered enthusiasm into organized community practice through residents’ committees, neighborhood centers, and civic groups. Small-scale municipal grants, rental subsidies, or time-slot access rights could activate local stewardship without requiring high capital inputs (25). Training volunteers as facilitators would reduce the personal burden on the government while increasing program continuity (5). Localizing education would mark a significant shift in structure (30). Introducing pickleball selectively into school curricula and teacher-training institutes would generate long-term behavioral imprinting and normalize activity among youth, a prerequisite for intergenerational diffusion (31). University-based certification for coaches and referees would create a qualified talent pipeline, enabling systematic rather than ad-hoc delivery (34, 61). To support public health goals, health departments and community clinics could promote pickleball as a way for older, inactive, or chronically ill people to stay healthy and function better (2). Linking sports participation with medical advice, rather than just branding it as a fun activity, would achieve this (27, 33). Digital platforms linking clubs, venues, and users could further institutionalize participation by reducing search costs, improving scheduling equity, and generating behavioral data useful for health governance (3, 44).

### 4.4 Competition system and integrated development localization

A localized competition structure would need to balance inclusivity with recognition effects (7, 8). Entry-level leagues in communities and campuses would sustain participation without professionalization pressure. Regional events organized under unified technical rules would lend administrative legitimacy and provide experiential reinforcement for participants (38). A national championship would serve primarily as a signaling device, demonstrating that pickleball has crossed the legitimacy threshold required for nationwide adoption. Professionalization elements, including certification systems, digital event management, and officiating standards, would serve governance and health functions simultaneously (28). These tasks would be done by stabilizing quality, reducing injury risk, and building public trust (62). Cross-sector integration would further increase its relevance and scale (45). Tourism departments could integrate pickleball into wellness-tourism portfolios, not as commercial spectacles alone but as participatory offerings aligned with healthy-aging agendas (40, 59). Health facilities could incorporate pickleball into organized rehabilitation programs for cardiovascular or musculoskeletal disorders, utilizing its low-impact design (41). Education and labor sectors could employ pickleball as a tool for student well-being and workplace wellness (1). Media collaboration would be required not for entertainment value but to embed the sport within narratives of active aging, social cohesion, and sustainable lifestyles. These frames align with population-health discourse rather than athletic prestige.

### 4.5 Synthesis: translating experience into localization mechanisms

**Figure 3:**
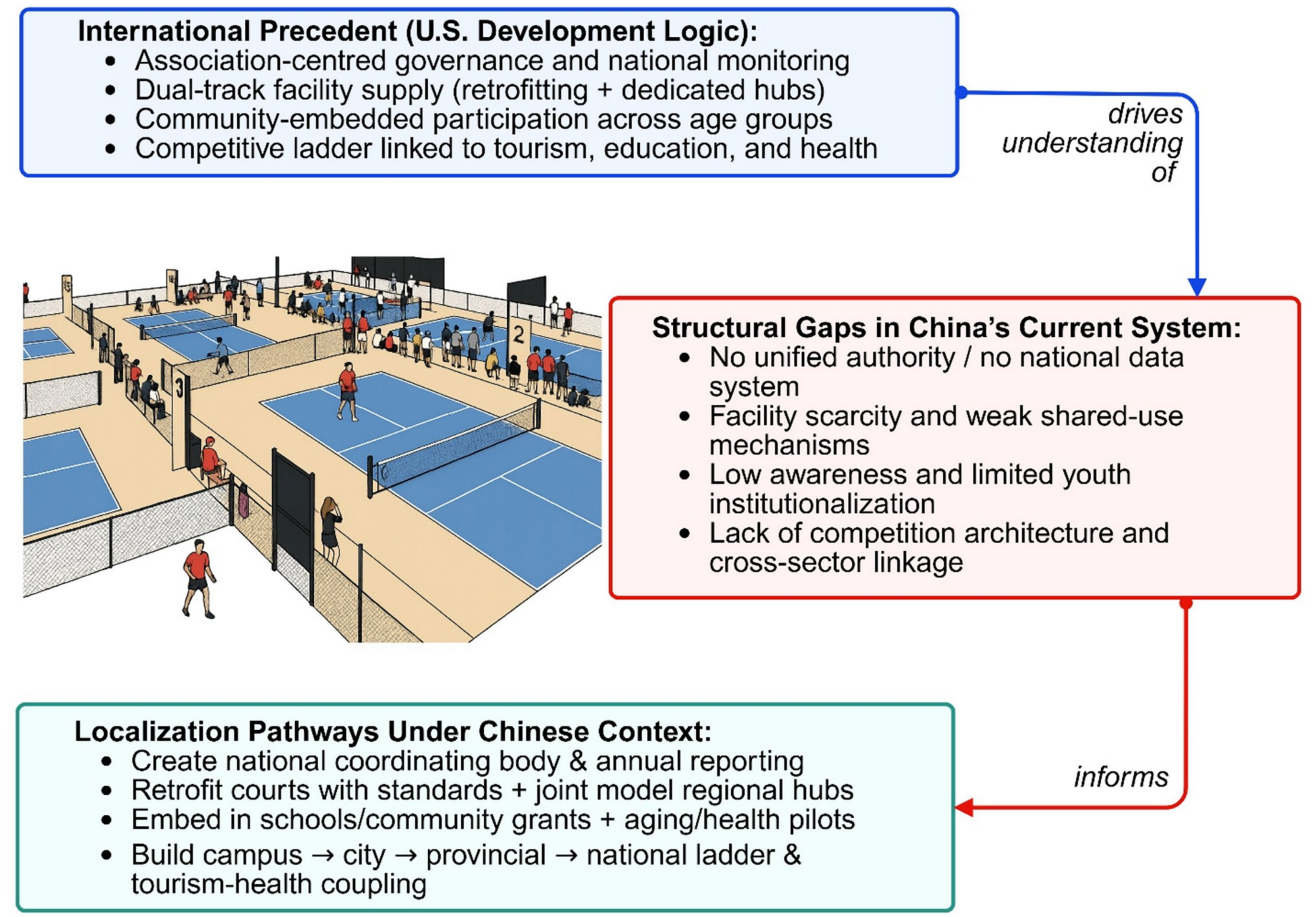
US governance reference, China constraints, and localization pathways for pickleball.

The US case illustrates that rapid diffusion emerged not from cultural affinity but from institutional conditions that reduced adoption costs, increased social normalization, and provided governance continuity (20). Translating that logic into China, it would prioritize mechanisms rather than models. Association-type governance under state recognition could create policy legitimacy without extinguishing civic initiatives (18). Evidence-based facility strategies would reduce fiscal and spatial barriers while aligned with equity-oriented planning (7, 8). Community cultivation and educational adoption would transform the sport into a recurring social practice rather than an episodic novelty (63, 64). Competition systems and cross-sector links would stabilize participation incentives, broaden their relevance, and justify resource allocation. Together, these pathways constitute a localization framework wherein pickleball functions as a vehicle for public-health promotion embedded within China’s administrative and social architecture. Localization is not an act of duplication but of institutional translation. The political feasibility of such translation depends on incremental implementation within existing administrative hierarchies, alignment with national strategic priorities, and phased pilot programs that demonstrate measurable outcomes before formal institutional expansion. By aligning governance functions with China’s policy environment and health objectives, pickleball could shift from scattered experimentation to structured integration into the national fitness system (65, 66). Such a transition would enable the sport to contribute to recreational diversification but also to the prevention of sedentary risk (54, 67). It would also extend active life expectancy, and facilitate the development of a community-based health infrastructure (9). The next section examines the policy implications and the conceptual contributions of this localization logic to broader discussions about sport-health governance. These mechanisms collectively form a coherent localization framework that balances top-down coordination with bottom-up participation (Figure 3).

## 5 Discussion

The comparative findings indicate that the transfer of pickleball development experience from the US to China is not only conceptually plausible but also practically feasible when approached as a process of functional translation rather than institutional replication. Although the two countries differ in governance architecture, regulatory logic, and cultural participation norms, the mechanisms that enabled pickleball to scale in the US, including association-led coordination, cost-efficient facilities, community embedding, and cross-sector integration, possess functional equivalents within China’s state-led, policy-driven sport system. The discussion below synthesizes feasibility conditions, enabling levers, and implementation considerations that shape the likelihood of successful localization in a way that strengthens population-health objectives rather than merely expanding a recreational niche.

This analysis is subject to several limitations. First, it relies primarily on secondary literature, policy documents, and publicly available reports rather than primary fieldwork or interview-based evidence from Chinese administrative institutions. As a result, interpretations of governance dynamics and participation patterns are inferential rather than ethnographically grounded. Second, the absence of standardized national data on pickleball participation in China limits the ability to provide precise quantitative comparisons. Third, the proposed localization pathways are analytical projections based on institutional logic rather than empirically evaluated pilot outcomes. Future research incorporating field studies, administrative interviews, and longitudinal participation data would strengthen the empirical foundation of the framework.

### 5.1 Institutional feasibility and governance transition

The establishment of a national-level authority responsible for standardization, monitoring, and strategic coordination appears institutionally achievable within the Chinese sport-governance framework, provided the process is sequenced rather than abrupt. Embedding pickleball governance initially under an existing racquet-sport federation could mitigate bureaucratic resistance while securing administrative legitimacy. This transitional model would enable early-stage regulation, accreditation, and reporting without necessitating immediate organizational independence (22). Ongoing reforms that promote “decoupling administration from management” in sports associations make this approach more likely to work (27). These reforms allow semi-autonomous entities to operate under government recognition. A dual-track governance model, which includes state-led at the strategic level and socially executed at the operational level (25), aligns with China’s recent direction toward “government guidance + social participation”. This structure would allow pickleball to mature organizationally while ensuring compliance, fiscal oversight, and quality control (30). From a public-health standpoint, institutional anchoring is critical because physical-activity interventions without governance support remain fragile and unevenly distributed.

### 5.2 Policy coordination and data infrastructure

The feasibility of evidence-based governance depends on establishing a minimum-viable data infrastructure rather than a fully mature system at its inception (28). A standardized reporting template for participation, venue availability, and demographic composition could be introduced in pilot provinces before the national rollout. Integrating such data into the National Fitness Information Platform would position pickleball within state-recognized evaluation frameworks (31), which is a prerequisite for receiving budgetary and planning support. Coordinated data systems also enable integration with health monitoring, making it possible to evaluate participation among seniors, sedentary groups, and chronic-disease-risk populations (2). This linkage strengthens the justification for policy support (18), as it connects sport development to measurable public-health outcomes rather than recreational preferences. Existing digital infrastructures for community fitness registration enhance the feasibility of building such systems at minimal marginal costs.

### 5.3 Infrastructure constraints and financial sustainability

The US experience suggests that expansion through adaptive reuse rather than new construction is a realistic strategy under urban land and budget constraints. In China, this approach aligns with current mandates encouraging the “opening of public facilities” and the use of modular, shared-use planning (11). The proposed dual-track facility strategy refers to a phased infrastructure model combining (1) low-cost adaptive retrofitting of existing courts to enable immediate and geographically distributed access, and (2) selective development of purpose-built facilities in areas with demonstrated demand to support formal instruction, structured programming, tournaments, and long-term operational sustainability. Local governments already possess procedural precedents for designating school and community facilities for dual use, reducing institutional uncertainty (20). Fiscal sustainability is strengthened when public investment acts as a catalyst and private capital supplements rather than replaces public provision. Public-private partnership models allow the government to subsidize accessibility while enabling developers to contribute infrastructure as part of residential or commercial projects (34). This arrangement creates incremental gains in facility supply without burdening public budgets (38). The feasibility of this approach is particularly relevant in older urban districts, where retrofitting adds functional value without new land acquisition. It is also relevant in rural regions, where modular courts can serve aging populations at low cost (17).

### 5.4 Social awareness, participation, and educational integration

The US case illustrates that participation scaled not because of cultural alignment but because playing opportunities were visible, normalized, and socially supported. In China, social acceptance can be advanced by aligning pickleball with national narratives such as Healthy China, active aging, social harmony, and low-cost recreation. Positioning the sport within these frames enables symbolic legitimacy before mass familiarity takes hold (3). Educational integration represents a high-leveraging, long-horizon mechanism (40). Introducing pickleball as an elective component of school or university curricula allows diffusion to become generational rather than episodic (41, 61). Teacher training and curricular inclusion require procedural time but once enacted, they produce durable cultural imprints (12). Meanwhile, community-based participation among older adults, reinforced by health institutions recommending pickleball as an appropriate moderate-intensity exercise, strengthens the activity’s association with preventative health rather than leisure consumption alone (7, 8, 50). These feasibility conditions suggest that social diffusion deepens when health, education, and community systems co-produce (10).

### 5.5 Cross-sector collaboration and economic integration

The feasibility of cross-sector collaboration depends not on structural similarity with the US but on functional complementarity with Chinese policy agendas. Pickleball integration with tourism, senior services, rehabilitation, and wellness programs becomes viable when framed as contributing to local public-value objectives rather than commercial expansion (45). For example, incorporating pickleball into wellness-tourism packages or community health campaigns can generate economic and social returns simultaneously without introducing prohibitive cost barriers (1). Interagency coordination is achievable through joint task forces or cooperation memoranda, especially when projects can demonstrate co-benefits across ministries. Pilot programs illustrating health cost savings or tourism revenue can justify shared funding pools and reduce siloed budgeting. Importantly, integration feasibility increases when economic effects are treated as secondary outcomes of a health-promoting system rather than as primary development goals (16). Such an approach reduces the risk of premature commercialization that could limit equitable access.

### 5.6 Implementation risks and enabling conditions

Localization feasibility does not eliminate this risk. Institutional inertia may delay formal recognition, resource asymmetry may disadvantage less developed regions, and cultural perceptions may initially frame pickleball as age-specific or novelty-based (4). However, these barriers are not absolute constraints but transitional phenomena that are addressable through sequencing and framing (5). Experimentalist governance, which starts with provincial pilots before national codification, lowers systemic risk and allows adaptive correction. Transparent documentation of pilot outcomes builds institutional learning and reduces uncertainty for later adopters. Prioritizing facility retrofitting in low-income areas and adding pickleball to state-funded senior and health programs before commercial offerings take over can reduce equity risks (7, 8). Cultural adaptation challenges diminish as the sport becomes visible in education, health, and media channels that confer symbolic legitimacy (33). Rapid expansion of emerging sport infrastructure may generate unintended consequences in dense urban environments. Adaptive court retrofitting can create space-use conflicts within multifunctional public facilities, particularly where demand exceeds allocation capacity. Scheduling and access priorities perceived as inequitable may lead to intergenerational and neighborhood tensions. Additionally, noise externalities and localized overuse can trigger community resistance. If expansion proceeds without calibrated access rules and conflict mediation mechanisms, emerging sport promotion may unintentionally exacerbate intergenerational tensions, privilege commercially viable districts over underserved neighborhoods, or concentrate benefits among socially active populations while excluding sedentary high-risk groups. At the policy level, there is also a risk that excessive framing of recreational sport solely as a health intervention may contribute to over-medicalization, potentially diminishing its intrinsic social and voluntary appeal. Recognizing these risks supports a more balanced institutional design and conflict-sensitive implementation.

### 5.7 Practical implications

Taken together, the analysis indicates that localization is feasible when approached as a staged institutional process anchored in governance legitimacy, evidence-based monitoring, spatial pragmatism, and cross-sector alignment (49). Short-term feasibility rests on establishing a governance home and data foundation; medium-term feasibility depends on facility integration and social embedding; long-term feasibility is achieved when the sport is not merely available but institutionally entangled with health, education, and community systems (9, 44). Under these conditions, pickleball could evolve from a grassroots experiment into a structural component of China’s national fitness and public-health architecture, delivering sustained benefits in population activity, active aging, social cohesion, and equitable access to low-cost sport participation.

## 6 Conclusion and policy implications

### 6.1 Conclusion

This study examined the divergent developmental trajectories of pickleball in the US and China to identify governance mechanisms relevant for localized diffusion. The US model is characterized by four interacting components: association-led coordination supported by systematic data monitoring; a dual-track facility strategy combining retrofitting with selective new construction; community-embedded participation across age groups and social settings; and a multi-tier competition system linked to tourism, wellness, and service-sector initiatives. Together, these elements transformed a low-threshold activity into a stable component of the public-activity landscape with measurable reach. In China, pickleball remains at an exploratory stage, constrained by governance fragmentation, absence of data systems, facility limitations, low public awareness, and weak integration with adjacent policy domains. These constraints, however, do not represent structural prohibitions. The analysis suggests that the US experience is transferable in function even if not replicable in form. Within a state-led governance environment, functional elements of the US model, which include centralized coordination, evidence-based planning, educational and community embedding, and cross-sector linkages, could be adapted to support population-level uptake. Reframing pickleball as a governance instrument for active aging, sedentary-risk reduction, and community vitality rather than as a recreational novelty provides both policy relevance and public-health significance. Theoretically, this study contributes to sport-health governance literature by demonstrating that the diffusion of emerging sports depends on transferable institutional functions rather than institutional replication, thereby advancing discussions of policy translation, adaptive governance, and functional equivalence in comparative public-health systems.

### 6.2 Policy implications

Practically, the analysis translates this functional framework into staged administrative actions that policymakers can adopt within existing governance structures to support scalable, equitable, and health-oriented sport diffusion. First, progressive institutionalization is essential for administrative visibility and coordination. A national governance anchor, either independent or embedded within an existing racquet-sport federation, could standardize rules, accredit personnel, authorize events, and publish annual development reports, enabling pickleball to enter formal planning and funding channels. Second, data infrastructure is expected to precede large-scale investment. Unified reporting templates incorporated into the National Fitness Information Platform would create standards for participation, demographic reach, and spatial distribution. Reliable metrics would prevent redundant construction and support differentiated, need-based promotion strategies across regions and populations. Third, facility planning is recommended to prioritize adaptive reuse and equity. Retrofitting existing school and community courts with standardized multi-use guidelines would expand access at a low cost. Co-investment models involving government, enterprises, and communities could balance feasibility and accessibility when new construction is necessary. Modular, low-cost facilities in rural areas would mitigate spatial inequality and align with healthy-aging policies beyond metropolitan centers. Fourth, participation growth depends on educational and community embedding. Pilot adoption in school curricula, university certification programs, and neighborhood fitness initiatives would generate stable user bases and intergenerational continuity. For older adults and health-sensitive groups, integration with rehabilitation centers, senior institutions, and chronic-disease-prevention programs would position pickleball as a health resource rather than a leisure option. Fifth, cross-sector alignments can amplify policy returns while preserving equity. Integration with wellness tourism, healthy-aging services, and community-care programs can diversify resources and broaden impact if commercialization does not constrain access. Joint coordination among sport, education, and health authorities could reduce policy silos, align incentives, and legitimize multi-source investment. Overall, a phased, risk-aware pathway, which includes institutional anchoring first, data construction second, spatial optimization third, social embedding fourth, and cross-sector integration last, provides a feasible route for transitioning pickleball from a grassroots initiative into a durable component of China’s national fitness and public-health infrastructure. When aligned with Healthy China and active-aging agendas, sport development contributes to long-term objectives of social cohesion, preventive health, and inclusive urban–rural well-being.

Future research may move beyond comparative institutional analysis to empirical evaluation of localized pilot programs. Longitudinal field studies examining participation patterns, health outcomes, cost-effectiveness, and equity impacts across different regions would provide stronger evidence for policy refinement. Mixed-method approaches incorporating administrative interviews, community-level case studies, and behavioral data analytics could clarify how governance sequencing influences adoption trajectories. Evaluating unintended consequences, including spatial conflicts, commercialization pressures, and differential access among vulnerable populations, will also be essential to ensuring that sport-health integration remains equitable and sustainable.
